# *RNF43* Mutations in IPMN Cases: A Potential Prognostic Factor

**DOI:** 10.1155/2020/1457452

**Published:** 2020-08-31

**Authors:** Xiao Yan Chang, Yan Wu, Ying Jiang, Peng Yan Wang, Jie Chen

**Affiliations:** Department of Pathology, Peking Union Medical College Hospital, Chinese Academy of Medical Sciences and Peking Union Medical College, Tsinghua University, Beijing 100730, China

## Abstract

An intraductal papillary mucinous neoplasm (IPMN) is a common pancreatic precursor lesion, and it often harbors mutations in *KRAS*, *GNAS*, and *RNF43*. To clarify the molecular profiles of IPMNs, we conducted mutation analysis of *KRAS*, *GNAS*, and *RNF43* in 61 IPMN formalin-fixed, paraffin-embedded (FFPE) specimens. The mutation rates of codons 12, 13, and 61 in *KRAS* and codon 201 in *GNAS* were detected by Sanger sequencing. Next-generation sequencing was performed on *RNF43*, and the results were further verified by Sanger sequencing. We identified *KRAS* and *GNAS* mutations in 35 (57%) and 40 (66%) IPMN cases, respectively. *GNAS* mutations were significantly correlated with the morphologic subtype (*P* < 0.001) and were more prevalent in the intestinal subtype (93%) than in the gastric (55%) and pancreatobiliary subtypes (44%) but were absent in the oncocytic subtype. *RNF43* mutations were found in 5 cases (8%), all of which occurred in high-grade dysplasia and invasive lesions (2/5 and 3/5). All 5 cases harboring RNF43 mutations also exhibited GNAS mutations. RNF43 mutations were associated with a worse prognosis in invasive IPMN patients (*P* = 0.002), while KRAS and GNAS mutations did not affect the prognosis of patients.

## 1. Introduction

An intraductal papillary mucinous neoplasm (IPMN) is a grossly detectable pancreatic precancerous lesion that can progress to invasive pancreatic cancer. Most likely due to the development of imaging technology, IPMN has become more commonly detected, comprising almost one-half of the surgically resected pancreatic cystic neoplasms; therefore, it is currently the most commonly resected cystic neoplasm of the pancreas [1]. IPMNs occur equally in men and women, with a mean age of 60 years [2]. Generally, approximately 23% of IPMN patients demonstrate high-grade and infiltrative IPMNs [3]. Tumors located in the main pancreatic duct have a significantly higher risk of malignant transformation than do those of the branch duct type [4]. The associated invasive component of IPMNs includes ductal, colloid, and oncocytic carcinoma, while the first two types have better prognosis than the third one [5]. Although there was no demographic discrepancy in invasive versus noninvasive IPMNs, the reported recurrence rates of surgically IPMNs are 12.1-100% and 0-12.9%, respectively [6].

In the second edition of the WHO classification, IPMN was defined for the first time as an independent entity, characterized by a cystic neoplasm with dimensions > 1 cm [7]. Histologically, IPMN forms papillary structures in pancreatic duct -lining mucin-producing neoplastic cells. In the latest version of the WHO classification [8], noninvasive IPMN was graded as low-, intermediate-, or high-grade dysplasia. In 2015, the Baltimore consensus meeting revised this 3-tiered classification and recommended incorporating the previously defined low and intermediate grades into low grade because they both possessed low malignant potential. Based on the lineage of epithelial lining cells, IPMNs are categorized as gastric, intestinal, pancreatobiliary, and oncocytic subtypes. Some studies have concluded that morphological subtypes and invasion are statistically related to the survival rates for IPMNs [9, 10].

Molecular studies have discovered a wide variety of genetic alterations in IPMNs. It is the consensus that *GNAS*, *KRAS*, and *RNF43* are the most prevalent mutant genes. The point mutation of *GNAS* in codon 201 is the most common and specific molecular phenomenon in IPMNs [11]. The activation of mutated *GNAS* encodes an activated *α* subunit of guanine nucleotide-binding protein (G-protein), which activates the GPCR pathway [12]. *KRAS* is another common mutated oncogene with a higher mutation rate in pancreatobiliary and gastric types than in other types. *KRAS* exhibits intrinsic GTPase activity and toggles between GTP-bound and GDP-bound state, while its base pair substitutions are detected mostly in codons 12, 13, and 61. The molecular underpinnings of this mutation result in a resistant status of GTP-bound *KRAS* to GTPase, independence of growth factors, and constitutive activation [13], thus promoting persistent MAPK/ERK, PI3K-AKT-mTOR, and RALGDS-RAL signaling downstream [14]. *RNF43*, as a transmembrane E3 ubiquitin ligase, downregulates the Wnt/*β*-catenin pathway by ubiquitinating the Wnt receptor frizzled; thereby, it exerts tumor-suppressor activity in the pathogenesis of IPMN [15]. Mutations of *RNF43* have been reported in several neoplasms, including colorectal cancer, endometrial cancer, and mucinous cystic neoplasms of the pancreas (MCN) [16, 17]. In 2011, an *RNF43* mutation was identified in IPMN by Wu et al. for the first time [16].

Although many studies have reported the molecular features of IPMN, a limited number of studies have examined the *RNF43* mutation profiles and their clinical significance in IPMN. Additionally, complete and detailed data on IPMN in the Chinese population are lacking. In this study, we detected mutations of *KRAS*, *GNAS*, and *RNF43* in a series of 61 IPMN cases in China. Furthermore, we analyzed the correlations between *RNF43* mutations and the prognosis of IPMN patients by the Kaplan-Meier survival analysis.

## 2. Materials and Methods

### 2.1. Patients and Tissue Samples

Formalin-fixed, paraffin-embedded (FFPE) tissue blocks of 61 IPMNs, diagnosed between 2013 and 2015, were retrieved from the archives of Peking Union Medical College Hospital in China. The slides of the included cases were all reviewed by two pancreatic pathologists in our department. We reviewed the electronic medical records to obtain the clinical information of all the patients. The follow-up information of enrolled patients was obtained by telephone.

### 2.2. DNA Extraction

Ten 5 *μ*m thick sections were each cut from FFPE tissue blocks. After deparaffinization with xylene, the tumor components of noninvasive or invasive IPMNs were dissected under microscopic guidance. Genomic DNA was extracted from the sections using a QIAamp DNA FFPE Tissue Kit (Qiagen Inc., Valencia, CA, USA).

### 2.3. Analyses of Mutations in *KRAS* and *GNAS*

Extracted DNA was then subjected to a PCR amplification of the target region of the *GNAS* gene encoding codon 201 and the *KRAS* gene containing codons 12, 13, and 61. PCR amplification was performed by initial denaturation at 94°C for 5 minutes, followed by 40 cycles of denaturation at 94°C for 30 seconds, annealing at 58°C for 30 seconds, and extension at 72°C for 30 seconds, followed by a 10-minute final extension at 72°C using Taq DNA polymerase (Takara Bio Inc., Otsu, Japan). The primers used are listed in Table 1. PCR products were subjected to agarose gel electrophoresis and extraction and then analyzed by direct sequencing using a 3730xl Genetic Analyzer (ABI; Thermo Fisher Scientific, Inc., Waltham, MA, USA).

### 2.4. Next-Generation Sequencing and Sanger Sequencing Validation of *RNF43*

We designed a multiplex PCR amplification system to enrich the target fragments of *RNF43*. One thousand nanograms of each DNA sample was used for PCR amplification, followed by the addition of a tag sequence. Using a MiSeq Benchtop sequencer (Illumina Inc., San Diego, CA, USA), bidirectional sequencing was achieved for the fragments targeting all protein-coding exons as well as 5′UTR, 3′UTR, and splicing sites of *RNF43*. Alignments of raw reads of each sample to the GRCh37/hg19 human reference genome were performed using BWA software [19], and the preliminary results were corrected by the GATK standard process, including local realignment around InDels and base recalibration. The SNP/InDel of each sample was analyzed by VarScan (http://varscan.sourceforge.net/) and GATK HaplotypeCaller (https://software.broadinstitute.org/gatk/best-practices/), respectively. Subsequently, all SNP/InDel loci calling by these two software were intersected and annotated using ANNOVAR (http://annovar.openbioinformatics.org/en/latest/).

Based on the results of next-generation sequencing of *RNF43*, we validated the mutation status of *RNF43* by Sanger sequencing. The primers used for validation are listed in Table 2.

### 2.5. Statistical Analysis

Statistical analyses were performed using SPSS statistical software, version 21. Comparisons of continuous variables were performed using Student's *t*-test. The associations between categorical variables were analyzed using Pearson's chi-square test or Fisher's exact test. The follow-up information was analyzed by the Kaplan-Meier survival analysis with the log-rank test. *P* values less than 0.05 were considered statistically significant (two-tailed).

## 3. Results

### 3.1. Clinicopathological Features

The clinicopathological characteristics of all 61 cases are summarized in Table 3. In the morphological classification of these 61 IPMNs, 29 (48%) were the intestinal type, 11 (18%) were the gastric type, 16 (26%) were the pancreatobiliary type, and 5 (8%) were the oncocytic type. In histological grading of noninvasive IPMNs, 25 (76%) showed low-grade dysplasia, and 8 (24%) showed high-grade dysplasia. Invasive lesions accounted for 46% of all the IPMN cases. We obtained prognostic information by telephone follow-up, and the overall survival rate of these patients was 69.9%.

### 3.2. Somatic Mutations and Clinicopathological Features

**Figure 1:**
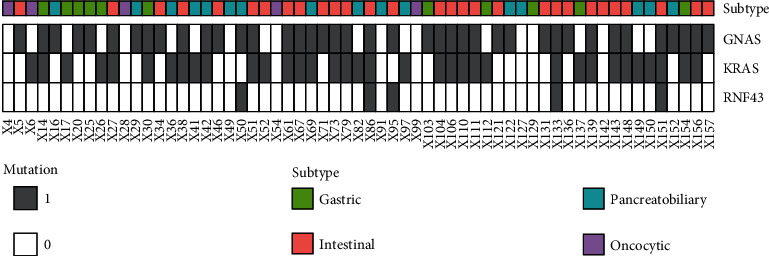
Mutation status of *KRAS/GNAS/RNF43* in 61 IPMNs.

**Figure 2:**
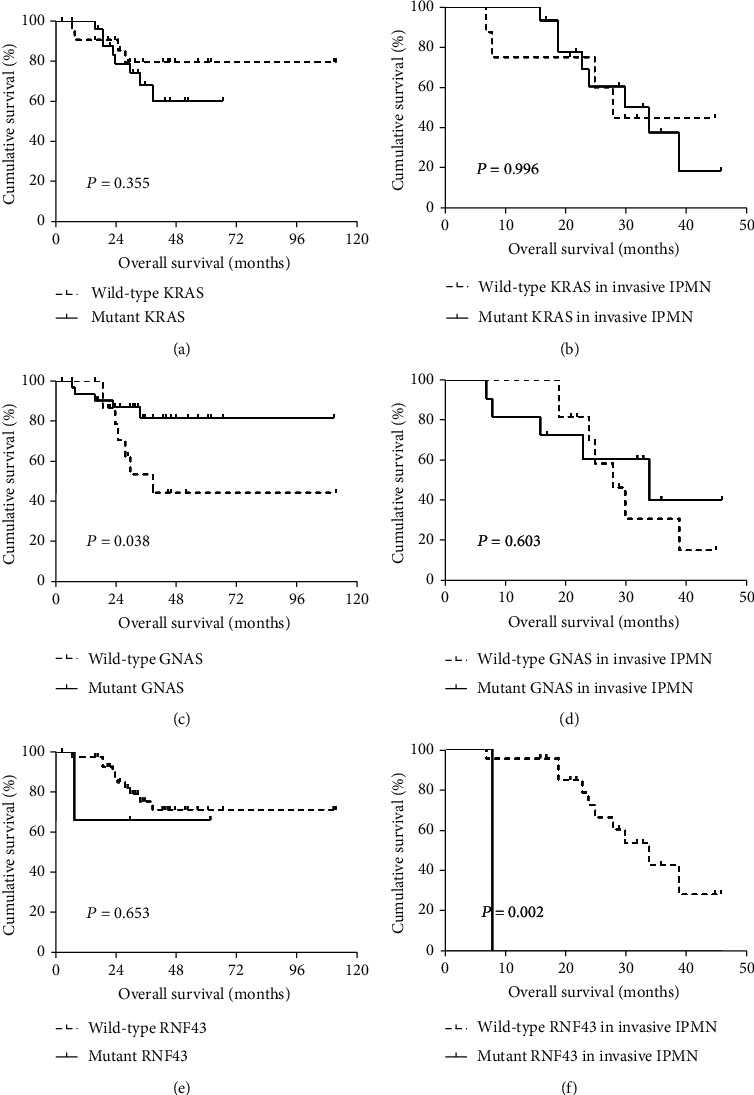
Kaplan-Meier survival analysis of IPMN patients based on the mutation status of *KRAS/GNAS/RNF43.*

*GNAS* mutations were detected in 40 of 61 (66%) IPMNs, and the following mutations were observed in codon 201: R201C in 22 tumors, R201H in 17 tumors, and R201Y in 1 tumor (Table 4, Figure 1, and Supplementary Table S1). The mean age of the *GNAS* mutated group was 65.1 years, which was significantly older than that of the nonmutated group (59.1 years) (*P* = 0.042, *t*-test). The *GNAS* mutation was significantly associated with the morphological type of IPMN (*P* < 0.001), and it was more common in the intestinal type (27/29, 93%) than in the gastric (6/11, 55%) and pancreatobiliary (7/16, 44%) types, but no mutations of *GNAS* were observed in any oncocytic (0/5, 0%) type. Our results also showed that nerve invasion was correlated with *GNAS* mutations (*P* = 0.029). In the 28 IPMN patients with invasive carcinoma, 6 exhibited nerve invasion. One of these 6 patients harbored a *GNAS* mutation (1/6, 17%), while 16 of the remaining 22 IPMN patients without nerve invasion harbored *GNAS* mutations (16/22, 73%). The nonnerve invasion group showed a much higher *GNAS* mutation frequency (Table 5). The Kaplan-Meier survival analysis indicated that *GNAS* mutation was correlated with survival time, and the prognosis of the *GNAS* mutation group was significantly better than that of the *GNAS* normal group (*P* = 0.038). Additionally, we performed a survival analysis in the invasive cohort. However, *GNAS* mutations did not impact the prognosis (*P* = 0.996) (Figure 2).

In this study, 8 of the 61 specimens (13%) contained mutations of the *RNF43* gene, including three in exon 9 (c.1093G>A: p.A365T), two in exon 7 (c.689delA: p.D230fs and c.700C>T: p.Q234X), one in exon 8 (c.879delG: p.E293fs), and two in splicing sites (exon4: c.253-2A>T and exon9: c.952+15A>G) (Table 4, Figure 1, and Supplementary Table S1). The p.A365T mutation of RNF43 is not present in relevant databases (COSMIC, ClinVar, and OncoKB), so we speculate that this may be due to polymorphism. As a result, the somatic mutation rate of RNF43 was 8% (5/61). All other 5 *RNF43* mutation cases were observed in the high-grade dysplasia group (*n* = 2) or the invasive group (*n* = 3), while no mutations of the *RNF43* gene were found in the low-grade dysplasia group. Albeit there was no significant association between *RNF43* mutation and the severity of dysplasia (*P* = 0.092), the high-grade group showed a higher mutation rate (22%) than did the low-grade group (0%) (Table 5). The survival curve seemed different between the *RNF43* wild-type group and the *RNF43* mutant group, and log-rank univariate survival analysis showed a significant difference in invasive IPMN patients (*P* = 0.002) (Figure 2).

*KRAS* was the second most commonly mutated gene, observed in 35 of 61 (57%) IPMNs. *KRAS* mutations were almost equivalent in gastric subtype (7/11, 64%), intestinal subtype (19/29, 66%), and pancreatobiliary subtype (8/16, 50%), but more frequent than the oncocytic type (1/5, 20%) (Figure 1 and Table 5). For the 35 cases harboring *KRAS* mutations, the mutation distribution was as follows: G12D (12/35, 34%), G12V (11/35, 31%), G12R (2/35, 6%), G12C (2/35, 6%), and G12N (1/35, 3%) (Table 4 and Supplementary Table S1). Regarding our data, *KRAS* mutation status did not appear to be correlated with any clinicopathological features. Survival analysis showed that the prognosis of patients was not associated with the mutation status of the *KRAS* gene in any of the IPMN patients or in the invasive IPMN patients (*P* = 0.355 and *P* = 0.996) (Figure 2).

Overall, the mutation of either *KRAS*, *GNAS*, or *RNF43* was found in 85% (52/61) of IPMNs, and 38% (23/61) had a mutation in both the *KRAS* and *GNAS* genes. Interestingly, all 5 cases harboring *RNF43* mutations also showed *GNAS* mutations, while there was no statistical significance (*P* = 0.092). And no correlation was observed between *GNAS* and *RNF43* mutations (*P* = 0.111) or between *KRAS* and *RNF43* mutations (*P* = 0.641).

## 4. Discussion

In this study, we detected the mutation rates of *GNAS*, *KRAS*, and *RNF43* in a series of 61 IPMNs in a single department in China, and we evaluated the correlations between their molecular abnormalities and clinicopathological features.

The *GNAS* mutation is a characteristic molecular alteration in IPMN that was reported by Wu et al. and Furukawa et al. for the first time [20, 21]. *GNAS* is located upstream of the GPCR pathway; therefore, the mutation of this gene might activate adenylyl cyclase and protein kinase A. The *GNAS* mutation frequency ranges from 30% to 79% and varies significantly among different histologic subtypes [12, 20–28]. Consistent with previous reports, our data also showed that the histological subtype of IPMNs had a significant impact upon *GNAS* mutational frequency [22]. The incidence of *GNAS* mutation was the highest (93%) in the intestinal type, followed by the gastric (55%) and pancreatobiliary (44%) types, and no mutations were found in the oncocytic type. This finding is also consistent with the report of Tan et al. that found that colloid-type invasive IPMN/colloid carcinoma possesses a large number of *GNAS* mutations because colloid carcinoma usually develops from intestinal-type IPMN [27]. The *GNAS* mutation group showed a better prognosis than did the wild-type group among all the IPMN patients; however, no difference was found in the prognosis between the *GNAS* wild-type and mutation groups among invasive IPMN patients. This inconsistency could be attributed to a higher proportion of infiltrative cases in the *GNAS* wild-type cohort (53%, 11/21) than in the mutation cohort (43%, 17/40), while infiltration is an important factor that affects the prognosis of IPMN patients.

*RNF43* encodes a ubiquitin E3 ligase that reduces the level of frizzled receptor and acts as a tumor suppressor in the Wnt signaling pathway. Unlike *KRAS* and *GNAS*, no definite hotspot exists for mutations of *RNF43*, which is characterized by nonsense mutations, missense mutations, or frame shift mutations that lead to a decrease in or loss of function [26]. The reported *RNF43* mutation rate was 12-18% in IPMN cases [23, 26–28], which was a little higher than our results (8%). Consistent with the results of Amato et al. [23], *RNF43* mutations were found only in the intestinal type (4/29, 14%) and pancreatobiliary type (1/16, 17%). In our study, the frequency of *RNF43* mutations was much higher in high-grade dysplasias (2/9, 22%) than that in low-grade dysplasias (0/24, 0%). We found all 5 cases harboring *RNF43* mutations also showed *GNAS* mutations. The patients harboring RNF43 mutation tend to have a worse prognosis, which need to be further validated in larger sample size.

*KRAS* mutations have been validated in most pancreatic preinvasive lesions, and they are considered an initiating step in the pathogenesis of pancreatic neoplasms [29]. Hotspot mutations in codon 12, 13, or 61 of *KRAS* are the most frequent events resulting in persistent MAPK/ERK signaling and other downstream pathway activation [14], and this crucial role of *KRAS* mutations has been proven using *KRAS^G12D^* mouse models [30–32]. Our results indicated that 35 of 61 (57%) cases displayed *KRAS* mutations, which was consistent with other reported results ranging from 32 to 81% [12, 20, 21, 23–28, 33]. Wu et al. showed that the pancreatobiliary subtype displayed the highest mutation frequency (100%), followed by the gastric (87%) and intestinal (46%) types [21]. Furukawa et al. also reported that the pancreatobiliary type had the highest *KRAS* mutation rate (67%), followed by the gastric (53%) and intestinal types (40%), but the difference was not statistically significant [20]. Our data showed that the intestinal, gastric, and pancreatobiliary types had a similar mutation rate, and the mutation pattern of *KRAS* was also the same as that in other reports. Among 35 *KRAS* mutation cases, 30 cases occurred in exon 12 (86%), of which G12D (34%) and G12V (31%) were the most common mutations.

There is a consensus that *KRAS* and *GNAS* mutations occur in the early stages of IPMN pathogenesis [34]. Consistent with the description of other studies, our data demonstrated no correlation between *KRAS/GNAS* mutations and tumor grade, and *KRAS* or *GNAS* mutation cases accounted for most of the IPMN cases (85%) in our cohort. Comparatively, *RNF43* occurs in the advanced disease stages, displaying high-grade dysplasia, suggesting that *RNF43* occurs in the progressive stage. Coincident with this conclusion, our survival analysis showed that the *RNF43* mutation was related to the prognosis of IPMN patients.

However, the sample size of IPMNs in our study was relatively small. We are continuing our efforts to expand the sample size of IPMNs and to detect additional genes. Although all *RNF43* mutations were identified in the cases harboring *GNAS* mutations which suggest the synergistic effect of *GNAS* and *RNF43* in the occurrence of IPMN, further research is required to determine the mechanism of action. The effect of *RNF43* mutation on prognosis of IPMNs also needs more cases to confirm. Further study of IPMNs is underway, and we intend to elaborate on the molecular mechanisms of this disease.

## 5. Conclusions

In summary, the results of *RNF43* analysis resulted in two essential findings regarding the tumorigenesis of IPMNs. First, *RNF43* mutations might have an effect in the progress of IPMN and influence the prognosis of patients. Second, based on the notably high frequency of *RNF43* mutations in high-grade and invasive lesions, we can infer that it may be possible for *RNF43* to distinguish between low-grade and high-grade dysplasia, which has implications for clinical diagnosis and treatment decisions.

## Figures and Tables

**Table 1:** PCR primers used for PCR amplification and Sanger sequencing.

Gene	Mutation site	Primer
*KRAS* [18]	Codons 12 and 13	Forward: AGGCCTGCTGAAAATGACTG
		Reverse: ATCAAAGAATGGTCCTGCAC
	Codon 61	Forward: TCCCTTCTCAGGATTCCTACA
		Reverse: CAAAGAAAGCCCTCCCCAGT
*GNAS*	Codon 201	Forward: AGACCTTTGCTTTAGATTGGC
		Reverse: CTTACTGGAAGTTGACTTTGTCC

**Table 2:** PCR primers used for validation of *RNF43* mutation.

Forward	Reverse	Chr start to end	Amplicon size
CTGCCCAATCAGGAATGCTAC	GAAGCCCGTGTATGGTATTTCA	58415234-58415771	538
CTGCCTCCACTACCTGTGTCC	AAACATGGGGACAAGAGAGCA	58370823-58371248	426
GACCCAGTGACCCCTTGCATA	TCACATGGGCCTTTTGGTTCT	58363304-58363757	454
CTGAGTGACTGGGAACGGAGT	AACTTCAGGCTGGTCCCTGAT	58360628-58361035	408
CTAAGGGAGTTGGGGGATGTC	TGGAGCAGTGTACAGCCCATA	58360009-58360335	327
TCCTTGATCCCCCAAACTCTG	TAGGCTGATGTCCGTGCAGTT	58358315-58358955	641
GGCTCTCTAACCCACAGTGC	CATCTCTGCTGTATCCTTCTCAGC	58357349-58357950	602
CACCTCCAATCCACCTCACAG	CCACTGGAACCGCTTTTTGTA	58358084-58358518	435

**Table 3:** Clinicopathological features of IPMN patients.

Clinicopathological features		*N* (%)
Age (years)	Mean	63.05 ± 9.55
Sex	Male	39 (64)
	Female	22 (36)
Pancreatitis		21 (34)
Size (cm)	Mean	3.96 ± 1.95
Location		
Head		44 (72)
Body and tail		12 (20)
Both		5 (8)
Imaging classification		
Main duct		36 (59)
Branch duct		15 (25)
Both		10 (16)
Subtype		
Gastric		11 (18)
Intestinal		29 (48)
Pancreatobiliary		16 (26)
Oncocytic		5 (8)
Dysplasia		
Low		25 (76)
High		8 (24)
Infiltration		
≤2 cm		17 (28)
2-≤4 cm		8 (13)
>4 cm		3 (5)
Differentiation of invasive carcinoma		
High		16 (26)
Middle		8 (13)
Low		4 (7)
Extent of invasive carcinoma		
Intrapancreatic		12 (20)
Extrapancreatic		16 (26)
Nerve invasion		6 (10)
Lymph node metastasis		5 (8)

**Table 4:** Mutations of *KRAS*/*GNAS/RNF43* identified in IPMN.

Gene	Mutation	Amino acid	*N*
*KRAS*	c.34G>C	p.G12R	2
	c.34G>T	p.G12C	2
	c.35G>A	p.G12D	12
	c.35G>T	p.G12V	11
	c.34G>A/35G>A	p.G12N	1
	c.38G>A	p.G13D	2
	c.182A>G	p.Q61R	5

*GNAS*	c.601C>T	p.R201C	22
	c.602G>A	p.R201H	17
	c.601C>T/c.602G>A	p.R201Y	1

*RNF43*	NM_017763:exon8:c.879delG	p.E293fs	1
	NM_017763:exon7:c.700C>T	p.Q234X	1
	NM_017763:exon7:c.689delA	p.D230fs	1
	NM_017763:exon4:c.253-2A>T		1
	NM_017763:exon9:c.952+15A>G		1
	NM_017763:exon9:c.1093G>A	p.A365T	3

**Table 5:** Correlation between clinicopathological features and *KRAS/GNAS/RNF43* mutation status in IPMN patients.

Clinicopathological features	*KRAS*				*GNAS*				*RNF43*			
	Wild type	Mutated	Mutated/total (%)	*P* value	Wild type	Mutated	Mutated/total (%)	*P* value	Wild type	Mutated	Mutated/total (%)	*P* value
No. of patients	26	35	35/61 (57)		21	40	40/61 (66)		56	5	5/61 (8)	
Age (mean, years)	62.3	63.6		0.625^☆^	59.1	65.1		0.042^☆¶^	63.1	62.8		0.952^☆^
Sex												
Male	17	22	22/39 (56)	0.839^†^	12	27	27/39 (69)	0.423^†^	35	4	4/39 (10)	0.645^△^
Female	9	13	13/22 (59)		9	13	13/22 (59)		21	1	1/22 (5)	
Pancreatitis												
Yes	8	13	13/21 (62)	0.604^†^	7	14	14/21 (67)	0.896^†^	21	0	0/21 (0)	0.154^△^
No	18	22	22/40 (55)		14	26	26/40 (65)		35	5	5/40 (13)	
Size (cm)												
≤3	12	12	12/24 (50)	0.376^†^	8	16	16/24 (67)	0.661^†^	22	2	2/24 (8)	1.000^△^
>3-≤5	8	17	17/25 (68)		10	15	15/25 (60)		23	2	2/23 (9)	
>5	6	6	6/12 (50)		3	9	9/12 (75)		11	1	1/12 (8)	
Location												
Head	19	25	25/44 (57)	0.692^△^	16	28	28/44 (64)	0.907^△^	40	4	4/44 (9)	0.263^△^
Body and tail	4	8	8/12 (67)		4	8	8/12 (67)		12	0	0/12 (0)	
Both	3	2	2/5 (40)		1	4	4/5 (80)		4	1	1/5 (20)	
Imaging classification												
Main duct	17	19	19/36 (53)	0.605^†^	15	21	21/36 (58)	0.316^†^	34	2	2/36 (6)	0.335^△^
Branch duct	6	9	9/15 (60)		3	12	12/15 (80)		14	1	1/15 (7)	
Both	3	7	7/10 (70)		3	7	7/10 (70)		8	2	2/10 (20)	
Subtype												
Gastric	4	7	7/11 (64)	0.261^△^	5	6	6/11 (55)	<0.001^△¶^	11	0	0/11 (0)	0.728^△^
Intestinal	10	19	19/29 (66)		2	27	27/29 (93)		25	4	4/29 (14)	
Pancreatobiliary	8	8	8/16 (50)		9	7	7/16 (44)		15	1	1/16 (6)	
Oncocytic	4	1	1/5 (20)		5	0	0/5 (0)		5	0	0/5 (0)	
Dysplasia												
Low	11	13	13/24 (54)	0.532^†^	7	17	17/24 (71)	0.819^†^	24	0	0/24 (0)	0.092^△^
High	5	4	4/9 (44)		3	6	6/9 (67)		7	2	2/9 (22)	
Infiltration												
Yes	10	18	18/28 (64)	0.315^†^	11	17	17/28 (61)	0.703^†^	25	3	3/28 (11)	0.421^△^
No	16	17	17/33 (52)		10	23	23/33 (63)		31	2	2/33 (6)	
Differentiation of invasive carcinoma												
High	7	9	9/16 (56)	0.339^△^	3	13	13/16 (81)	0.054^△^	13	3	3/16 (19)	0.464^△^
Intermediate	1	7	7/8 (87.5)		5	3	3/8 (38)		8	0	0/8 (0)	
Low	2	2	2/4 (50)		3	1	1/4 (25)		4	0	0/4 (0)	
Extent of invasive carcinoma												
Intrapancreatic	4	8	8/12 (67)	0.589^†^	3	9	9/12 (75)	0.295^†^	11	1	1/12 (8)	0.824^△^
Extrapancreatic	6	10	10/16 (63)		8	8	8/16 (50)		14	2	2/16 (13)	
Nerve invasion												
Yes	0	6	6/6 (100)	0.082^△^	5	1	1/6 (17)	0.029^△¶^	6	0	0/6 (0)	0.637^△^
No	10	12	12/22 (55)		6	16	16/22 (73)		19	3	3/22 (14)	
Lymph node metastasis												
Yes	2	3	3/5 (60)	1.000^△^	3	2	2/5 (40)	0.505^△^	5	0	0/5 (0)	0.770^△^
No	8	16	16/24 (67)		8	16	16/24 (67)		21	3	3/24 (13)	
Correlation between status of *KRAS* and *GNAS*												0.979^†^
Correlation between status of *KRAS* and *RNF43*												0.641^△^
Correlation between status of *GNAS* and *RNF43*												0.111^△^

^☆^ *t*-test. ^†^Chi-square test. ^△^Fisher's exact test. ^¶^Statistically significant (*P* < 0.05).

## Data Availability

All the data generated or analyzed during this study are included in this published article and its supplementary information files.

## Abbreviations

IPMN: Intraductal papillary mucinous neoplasm of the pancreas
KRAS: Kirsten rat sarcoma viral oncogene homolog
GNAS: Guanine nucleotide-binding protein, alpha stimulating
RNF43: Ring finger protein 43.

## Supplementary Materials

Supplementary Materials Table S1: the mutation sites of KRAS/GNAS/RNF43in IPMN cases.
