# Supplementary material for: RNF43 Mutations in IPMN Cases: A Potential Prognostic Factor
Source: Gastroenterol Res Pract. 2020 Aug 31;2020:1457452. doi: 10.1155/2020/1457452 (PMC7479465; doi:10.1155/2020/1457452)
Supplement: Supplementary Materials — Table S1: the mutation sites of KRAS/GNAS/RNF43in IPMN cases. [file 1457452.f1.docx]

| Table S1 The Mutation site of *KRAS*/*GNAS/RNF43* in IPMN cases | | | | |
| --- | --- | --- | --- | --- |
| Case | GNAS Condon201 | KRAS Condon12 | KRAS Condon61 | RNF43 |
| 4 | WT | WT | WT | WT |
| 5 | 602G>A | WT | WT | WT |
| 6 | WT | 34G>A/35G>A | WT | WT |
| 14 | 601C>T | 35G>T | WT | WT |
| 16 | 601C>T | WT | WT | WT |
| 17 | WT | 35G>T | WT | WT |
| 20 | 601C>T | WT | WT | WT |
| 25 | 602G>A | WT | WT | WT |
| 26 | WT | 35G>T | WT | WT |
| 27 | 601C>T | 35G>T | WT | WT |
| 28 | WT | WT | WT | WT |
| 29 | 601C>T/602G>A | WT | WT | WT |
| 30 | 601C>T | 35G>A | WT | WT |
| 34 | 601C>T | WT | WT | WT |
| 36 | WT | 35G>A | WT | WT |
| 38 | 602G>A | 34G>T | WT | WT |
| 41 | WT | WT | 182A>G | WT |
| 42 | 601C>T | 35G>A | WT | WT |
| 46 | 601C>T | WT | WT | WT |
| 49 | WT | WT | WT | WT |
| 50 | 602G>A | WT | WT | NM_017763:exon8:c.879delG |
| 51 | 601C>T | 35G>T | WT | WT |
| 52 | WT | 34G>C | WT | WT |
| 54 | WT | WT | WT | WT |
| 61 | 601C>T | 34G>T | WT | WT |
| 67 | 601C>T | WT | 182A>G | WT |
| 69 | 601C>T | 35G>T | WT | WT |
| 71 | 601C>T | WT | WT | WT |
| 73 | 602G>A | WT | 182A>G | WT |
| 79 | 601C>T | 35G>A | WT | WT |
| 82 | WT | 35G>A | WT | WT |
| 86 | 602G>A | 35G>T | WT | NM_017763:exon9:c.952+15A>G |
| 91 | WT | WT | WT | WT |
| 95 | 602G>A | WT | WT | NM_017763:exon4:c.253-2A>T |
| 97 | WT | 35G>T | WT | WT |
| 99 | WT | WT | WT | WT |
| 103 | 602G>A | WT | WT | WT |
| 104 | 602G>A | 35G>A | WT | WT |
| 106 | 602G>A | 35G>A | WT | WT |
| 110 | 602G>A | 38G>A | WT | WT |
| 111 | 601C>T | 35G>A | WT | WT |
| 112 | WT | 35G>T | WT | WT |
| 121 | 601C>T | WT | WT | WT |
| 122 | 601C>T | WT | WT | WT |
| 127 | WT | WT | WT | WT |
| 129 | WT | WT | WT | WT |
| 131 | 602G>A | WT | WT | WT |
| 133 | 602G>A | 35G>T | WT | NM_017763:exon7:c.689delA |
| 136 | 602G>A | WT | WT | WT |
| 137 | WT | 35G>T | WT | WT |
| 139 | 601C>T | WT | 182A>G | WT |
| 142 | WT | WT | WT | WT |
| 143 | 601C>T | 38G>A | WT | NM_017763:exon9:c.1093G>A |
| 148 | 601C>T | 35G>A | WT | WT |
| 149 | WT | 35G>A | WT | WT |
| 150 | WT | WT | 182A>G | WT |
| 151 | 601C>T | 35G>A | WT | NM_017763:exon7:c.700C>T |
| 152 | 601C>T | WT | WT | NM_017763:exon9:c.1093G>A |
| 154 | 602G>A | 35G>A | WT | WT |
| 156 | 602G>A | 34G>C | WT | WT |
| 157 | 602G>A | WT | WT | NM_017763:exon9:c.1093G>A |
